# Vaping Is Not Safe: A Case of Acute Eosinophilic Pneumonia following Cannabis Vapor Inhalation

**DOI:** 10.1155/2020/9496564

**Published:** 2020-01-27

**Authors:** Daniel Antwi-Amoabeng, Raheel Islam

**Affiliations:** Department of Medicine, University of Nevada Reno School of Medicine, Reno, NV, USA

## Abstract

There is a well-established association between inhalational exposures and acute eosinophilic pneumonia (AEP). The most reported exposure is cigarette smoking. Here, we present a case of progressive shortness of breath and nonproductive cough in a college student with no significant medical history, approximately 10 days after inhaling cannabis aerosols on two separate occasions. He was started on empiric antibiotics and bronchodilators without improvement. He was diagnosed with AEP based on peripheral eosinophilia and high-resolution CT image results. He made rapid recovery on intravenous glucocorticoids. Vaping has gained popularity among young adults mainly due to the perception that it is a safe alternative to smoking. This case shows that there may be a false sense of security with vaping. Vaping poses a yet-to-be quantified public health threat, which requires further studies.

## 1. Introduction

Acute eosinophilic pneumonia (AEP) is a rare cause of acute hypoxic respiratory failure. The exact pathophysiology is unknown, but it is thought to involve activation of the inflammation cascade resulting in recruitment of inflammatory cells predominantly, eosinophils to the lung parenchyma [1]. There have been reports associating this disease entity with exposure to drugs, dust, and cigarette smoking [2–6]. It generally occurs in men aged 20–30 years [7]. Patients present with nonspecific symptoms typical of community acquired pneumonia, and initial management is often focused on antibiotic therapy. Acute hypoxic respiratory failure, in an otherwise healthy young adult, can present a diagnostic challenge. Here, we present a case of acute eosinophilic pneumonia in a young male following cannabis inhalation.

## 2. Case Report

A 20-year-old male college student presented with three-week history of nonproductive cough, dyspnea, and wheezing. He was seen in the primary care office two weeks earlier and was started on albuterol and inhaled corticosteroids. He did not receive antibiotics. His symptoms briefly improved but he became more fatigued and, a week later, even more short of breath. He presented to the urgent care clinic and was found to be hypoxic with oxygen saturation of 83% on room air.

The patient's roommate had mild upper respiratory tract symptoms a few days prior to the onset of the patient's symptoms. The patient did not have fever, chills, headaches, myalgias or chest pain. Nor did he report engaging in risky sexual behavior or recent exotic travel. He denied cigarette smoking but admitted to smoking marijuana a few times a month. The patient recently started vaping marijuana, with first use approximately 10 days prior to onset of symptoms. He had vaped marijuana on two separate occasions prior to symptom onset. He had a history of allergic rhinitis and took antihistamines as needed. His father had childhood asthma; and there was no other significant family history. The patient was allergic to penicillin.

**Figure 1:**
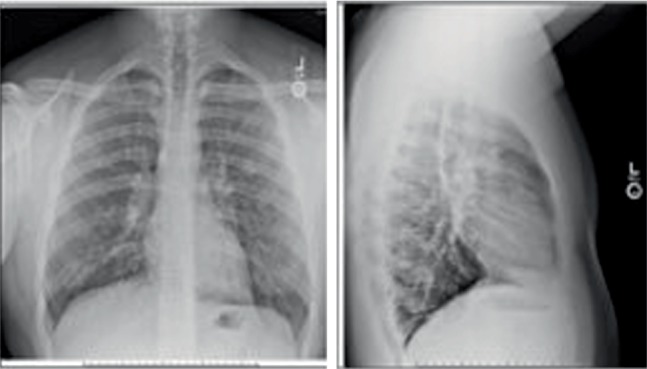
Two-view chest X-ray: increased pulmonary markings and possible multi-lobar pneumonia.

On admission, he was afebrile, blood pressure was normal, but he was tachycardic. He was taking 29 breaths per minute and needed 3 liters per minute (LPM) of oxygen by nasal canula to maintain an oxygen saturation of 89%. He appeared tired, but his breathing was unlabored. He had scattered wheezes on lung auscultation. Chest X-ray showed increased pulmonary markings and possible multi-lobar pneumonia (Figure 1). Complete metabolic panel was normal. WBC count was 35,000/*µ*L, neutrophil 20.7%, eosinophil 64.9% (21.77 K/*µ*L). His serum IgE was 823 kU/L. Tests for influenza A and B were negative. There was concern for community acquired pneumonia and he was started on Azithromycin and Ceftriaxone on admission but discontinued on the following day. We also considered allergic asthma, Allergic Bronchopulmonary Aspergillosis (ABPA), Eosinophilic Granulomatosis with Polyangiitis (EGPA), and other hypereosinophilic syndromes as potential etiologies for the patient's symptoms.

**Figure 2:**
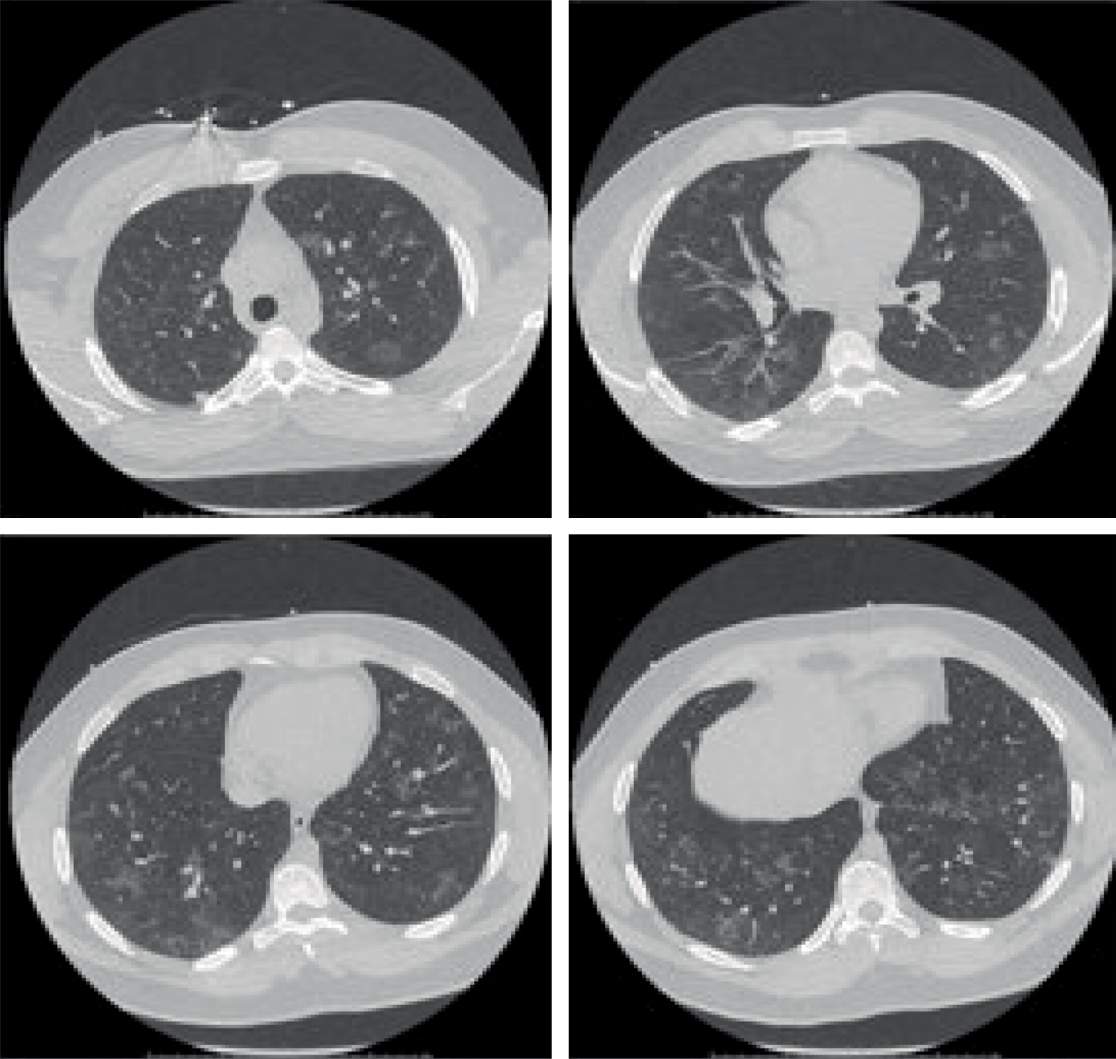
HRCT showing multifocal ill-defined ground glass opacities throughout both lungs.

He received nebulized bronchodilator treatment and oral Prednisone on admission, but his oxygen requirement had not improved on the next day. He needed 7 liters per minute oxygen via face mask to maintain oxygen saturation of 92%. In consultation with a Pulmonologist, antibiotics were discontinued due to strong suspicion of AEP on day one of hospitalization. High resolution CT of chest showed multifocal ill-defined ground glass opacities throughout both lungs, concerning for multifocal pneumonitis (Figure 2). The patient was started on Methylprednisolone 125 mg every eight hours, with rapid improvement in the eosinophilia and his respiratory status. A decision was made not to perform a bronchoalveolar lavage (BAL), because of his rapid response to corticosteroids and continued improvement in supplemental oxygen requirements. He was discharged home two days later with 2 LPM supplemental oxygen, steroid taper, and advised to desist from using marijuana in any form. Blood and sputum cultures remained negative on the day of discharge. Test for infectious etiology including HIV, Coccidioides, *Aspergillus*, Strongyloides antibody were all negative.

On follow-up, six days post discharge, he no longer required supplemental oxygen and had returned to his usual activity such as going to the gym. He saw an allergist three months later due to concern for ABPA. IgE had improved to 689 kU/L. Test for Anti-myeloperoxidase antibody, Protease 3b (PR3) antibody, antinuclear antibodies (ANA), Antineutrophil cytoplasmic antibody (ANCA) were all negative. Based on these results, EGPA was an unlikely etiology of his dyspnea and peripheral eosinophilia. He showed increased allergic response to cat demander, mites, *Aspergillus fumigatus*, and to tree and grass allergens. Tryptase assay returned normal result which, made a diagnosis of eosinophilic leukemia less likely. BAL at that time showed 75% eosinophils and the transbronchial biopsy showed pulmonary eosinophilia (Loeffler's type). Further, there were no parasites; no fungi; no granulomas; no organizing pneumonia; and no malignant cells in the BAL and biopsy samples. Bone marrow biopsy did not show increased blast cells, and no monotypic blast cell increase was observed either. The patient stopped using the vaping device. Unfortunately, he returned to smoking marijuana (via combustion) a few times with return of dyspnea and he needed prolonged steroid therapy.

## 3. Discussion

AEP is an uncommon cause of acute hypoxic respiratory failure and is associated with smoking cigarettes and/or cannabis [4, 8, 9]. In the present case, we report acute eosinophilic pneumonia in a patient following inhalation of aerosolized cannabis a few days prior to symptom onset. Further, we excluded infectious etiology as detailed above. ABPA was excluded by negative *Aspergillus* skin test, radiographic findings, and biopsy analysis. We also excluded acute eosinophilic leukemia by demonstrating normal bone marrow, a normal Tryptase assay, and the absence of anemia and thrombocytopenia on venous blood samples. He was not on any medications associated with drug reactions and eosinophilia. He did not have serologic or biopsy features typical of EGPA. He did have a history of atopy and rhinitis, which made allergic/atopic etiology a strong consideration, but his pulmonary infiltrates suggested a more diffuse etiology affecting the lung parenchyma. Since the patient had not changed the frequency or intensity of his marijuana smoking, we concluded that the recent initiation of cannabis vapor inhalation (vaping) was the precipitating factor in this case.

There is no consensus on the diagnostic criteria for AEP. The modified Philit Criteria [1, 10, 11] used to diagnose AEP include: (1) Acute respiratory illness of less than 1-month duration. (2) Pulmonary infiltrates on chest imaging. (3) BAL with eosinophils >25% or eosinophilic pneumonia on lung biopsy. (4) Absence of other eosinophilic pulmonary diseases, including eosinophilic granulomatosis with polyangiitis, hypereosinophilic syndromes; and allergic bronchopulmonary aspergillosis. Our patient met each of the diagnostic criteria for AEP. He had rapid improvement in clinical, laboratory and imaging findings after initiation of steroids; in line with prior reports [12, 13]. It appears re-exposure to precipitants in the immediate convalescence period that resulted in re-occurrence of symptoms and radiologic findings [13]. Although recurrence of AEP once a patient resumes smoking is not common, there have been some reports [14]. Our patient stopped vaping marijuana, but he returned to smoking marijuana a few times a week with recurrence of dyspnea and cough. Of critical note, his disease had progressed to chronic eosinophilic pneumonia on six-months follow-up. He appears to now depend on steroids as his symptoms came back when he stopped taking steroids.

The patient presented with features uncommon in AEP. First, he did not have a fever. Fever is often reported but not always present in those with AEP [4, 15, 16]. The modified Philit criteria removes “febrile illness” as a criterion. Thus, the absence of fever does not invalidate a diagnosis of AEP in this patient. Second, he had increased peripheral eosinophilia. Peripheral eosinophilia is not a prominent feature of AEP, but when present, it is associated with milder symptoms and may be a good prognostic feature useful in stratifying patients on initial presentation [17].

History of atropy may not be relevant to developing AEP, but total IgE levels and peripheral eosinophilia may correlate with disease severity [18]. The patient's serum IgE levels were markedly elevated when he initially presented, as in another reported case, [8] pointing to a role of type 1 hypersensitivity in the etiology of AEP. The levels were still high on follow-up but were lower than initial levels. His symptoms were resolved at that time as he was on steroids. E-cigarettes or vaporizers have become very popular. They are marketed as a substitute for cigarettes, and have been shown to be more effective for smoking cessation than traditional nicotine replacement therapy among adults [19]. These vaporizers have been adapted to deliver liquid and oil forms of tetrahydrocannabinol (THC) and cannabidiol (CBD) with a myriad of flavoring options [20]. The prevalence of cannabis vaping is speculative, but the act is perceived as innocuous, which may attract even the more cautious users. Youth describe the contents of vaping oils as harmless “water vapor,” which may be “good” for them [21].

The vapor from these vaping devices may contain particulate matter, as well as chemicals such as carbonyls, volatile organic compounds, and toxic metals, which have been linked to dyspnea, cough, upper airways irritation, asthma, pneumonia and lung cancer [22, 23]. Inhaling aerosols has been shown to increase airway resistance and decrease FEV1 in healthy nonsmokers [24].

Flavored vaping e-liquids have been found to contain high levels of diacetyl, [25] a substance known to be strongly associated with bronchiolitis obliterans syndrome in chemical workers producing diacetyl for food flavorings [26]. Diacetyl has been found in many flavored products including vaping liquids [27]. We suggest that the flavoring components, or components of the cannabis product, present an antigenic challenge to alveolar macrophages with exaggerated secondary response on subsequent exposure. The Centers for Disease Control and Prevention (CDC) has identified a new pulmonary pathology in patients who presented with insidious onset of dyspnea after recent use of THC-containing vaping products: e-cigarette, or vaping, product use associated lung injury (EVALI). In a recent update the CDC reports that Vitamin E acetate, used as a thickening agent, has been identified in BAL samples from several patients diagnosed with EVALI. THC was also identified in most of the samples from patients. In the same update, CDC reported that EVALI occurred mostly in males ranging in age from 13 to 78 [28]. It is impossible to determine the underlying pathophysiology of AEP in our patient. Certainly, further studies are required to outline the disease mechanisms involved in EVALI. For now, our best defense is public health education to discourage use of vaping products especially. Limiting vaping liquid flavors and the use of Vitamin E acetate in vaping products may help curb the popularity of vaping among youth and young adults and help avert a looming public health crisis of severe pulmonary illness in those who vape.

## 4. Conclusion

There have been recent reported cases of pulmonary illness associated with vaping. There have been prior reports of AEP associated with smoking marijuana. This case represents yet another example of the harms of vaping, which had, until recently, been unknown to the general public. We are unable to state if the specific THC product vaped by our patient contained vitamin E acetate, diacetyl or other potentially harmful additives. We have however, established a correlation between vaping cannabis aerosols and the onset of acute eosinophilic pneumonia in this patient. Acute hypoxic respiratory failure in an otherwise healthy young adult can present a diagnostic challenge, but peripheral eosinophilia, although an uncommon feature, and recent inhalational exposures should present early clues to suspect AEP. Prompt initiation of steroids is essential with rapid and complete resolution of symptoms, but symptoms may reoccur if the patient resumes exposure behavior during the convalescent period. Vaping behavior is very common among the youth and poses a preventable risk for debilitating pulmonary disease. Providers should discourage vaping in young nonsmokers and advise smokers to not take up vaping as a smoking cessation tool. They should also stick with conventional smoking cessation tools for smokers who wish to stop smoking cigarettes.
